# Supplementary material for: Multidimensional analyses reveal modulation of adaptive and innate immune subsets by tuberculosis vaccines
Source: Commun Biol. 2020 Oct 9;3:563. doi: 10.1038/s42003-020-01288-3 (PMC7547090; doi:10.1038/s42003-020-01288-3)
Supplement: Supplementary file 1 — Supplementary Information [file 42003_2020_1288_MOESM1_ESM.pdf]

Supplementary Material

Multidimensional analyses reveal modulation of adaptive and innate immune subsets by tuberculosis vaccines

Virginie Rozot[a#*], Elisa Nemes[a#], Hennie Geldenhuys[a], Munyaradzi Musvosvi[a], Asma Toefy[a], Frances Rantangee[a], Lebohang Makhethe[a], Mzwandile Erasmus[a], Nicole Bilek[a], Simbarashe Mabwe[a], Greg Finak[b], William Fulp[b], Ann M. Ginsberg[c], David A. Hokey[c], Muki Shey[d], Sanjay Gurunathan[e], Carlos DiazGranados[e], Linda-Gail Bekker[f], Mark Hatherill[a], Thomas J. Scriba[a]* and the C-040-404 Study Team

[a]South African Tuberculosis Vaccine Initiative, Institute of Infectious Disease & Molecular Medicine and Division of Immunology, Department of Pathology, University of Cape Town, Cape Town, South Africa; [b]Fred Hutchinson Cancer Research Center (FHCRC), Seattle, USA; [c]AERAS, Rockville, Maryland, USA; [d]Aeras South Africa Endpoint Assay Laboratory, Cape Town, South Africa; [e]Sanofi Pasteur, Swiftwater, Pennsylvania, USA; [f]The Desmond Tutu HIV Centre, University of Cape Town, Cape Town, South Africa.

[#] Equal contribution

*Corresponding authors

Supplementary Figure 1: Correlations between WBA and PBMCs.

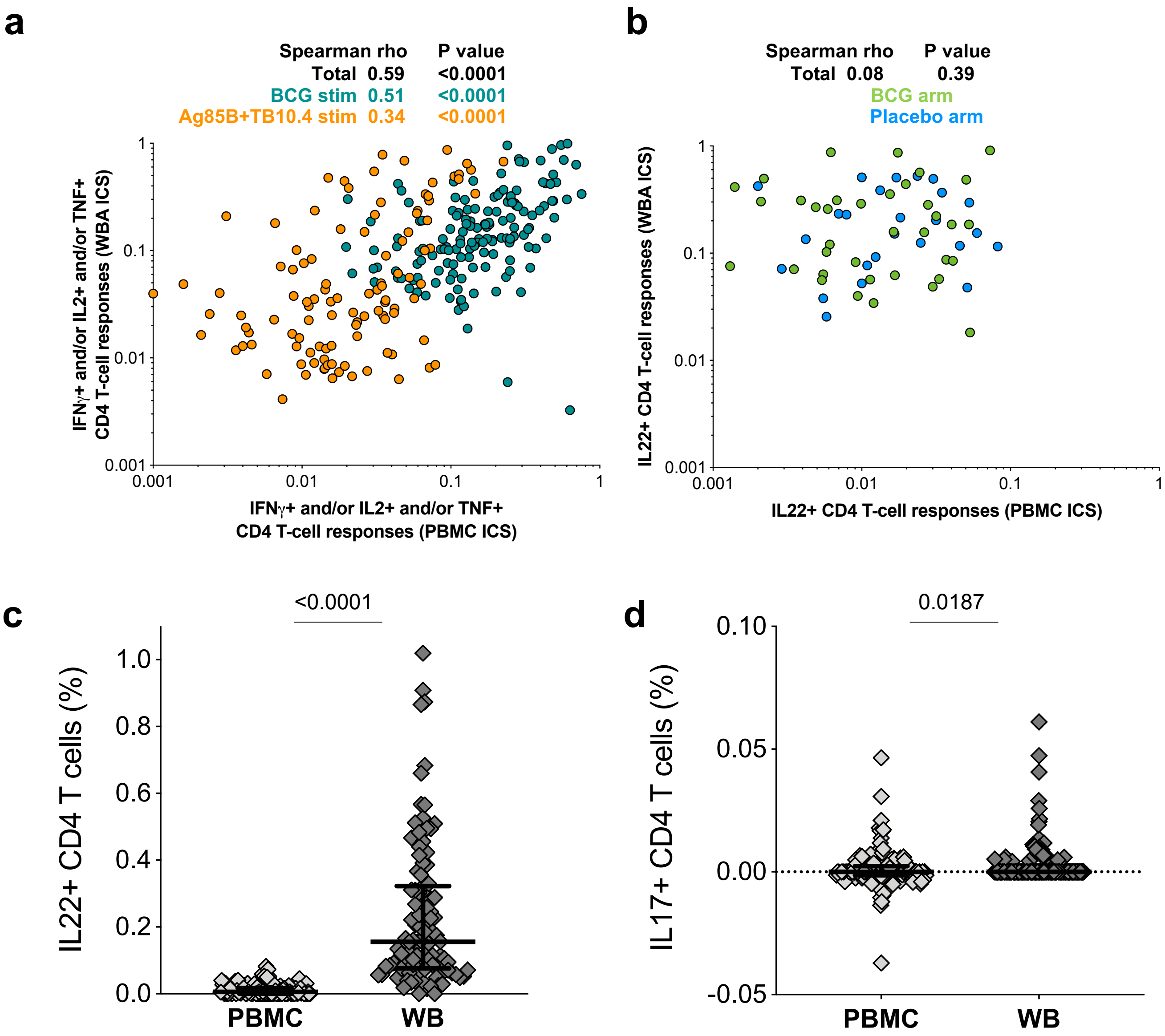

(a) Correlation of Th1 (IFNg+, IL-2+ and/or TNF+) CD4 T cell responses measured at day 0 and day 70 in the three vaccine arms after 12 hours stimulation with either Ag85B, TB10.4 or BCG by whole blood or PBMC Intracellular Cytokine Staining (ICS) assay. BCG stimulated samples are shown in dark green and Ag85B and TB10.4-stimulated samples in orange. Spearman correlation coefficients and p-values are shown for the total dataset. **(b)** Correlation of Th22 CD4 T cell responses measured at day 0 and day 70 in the placebo (blue) and BCG (green) vaccine arms after 12 hours stimulation with BCG by whole blood or PBMC Intracellular Cytokine Staining (ICS) assay. There were no Th22 responses detected in response to Ag85B and TB10.4 stimulations. Spearman correlation coefficients and p values are shown for all responses. **(c)** Frequencies of IL-22+ CD4 T cells observed in PBMC and WB ICS assays by Mann-Whitney test at day 0 and day 70 in placebo and BCG arms in response to BCG stimulation. **(d)** Frequencies of IL-17+ CD4 T cells observed in PBMC and WB at day 70 in H4:IC31 and BCG arms in response to Ag85B, TB10.4 and BCG stimulations. Comparisons are performed by Mann Whitney test.

Supplementary Figure 2: Gating strategy.

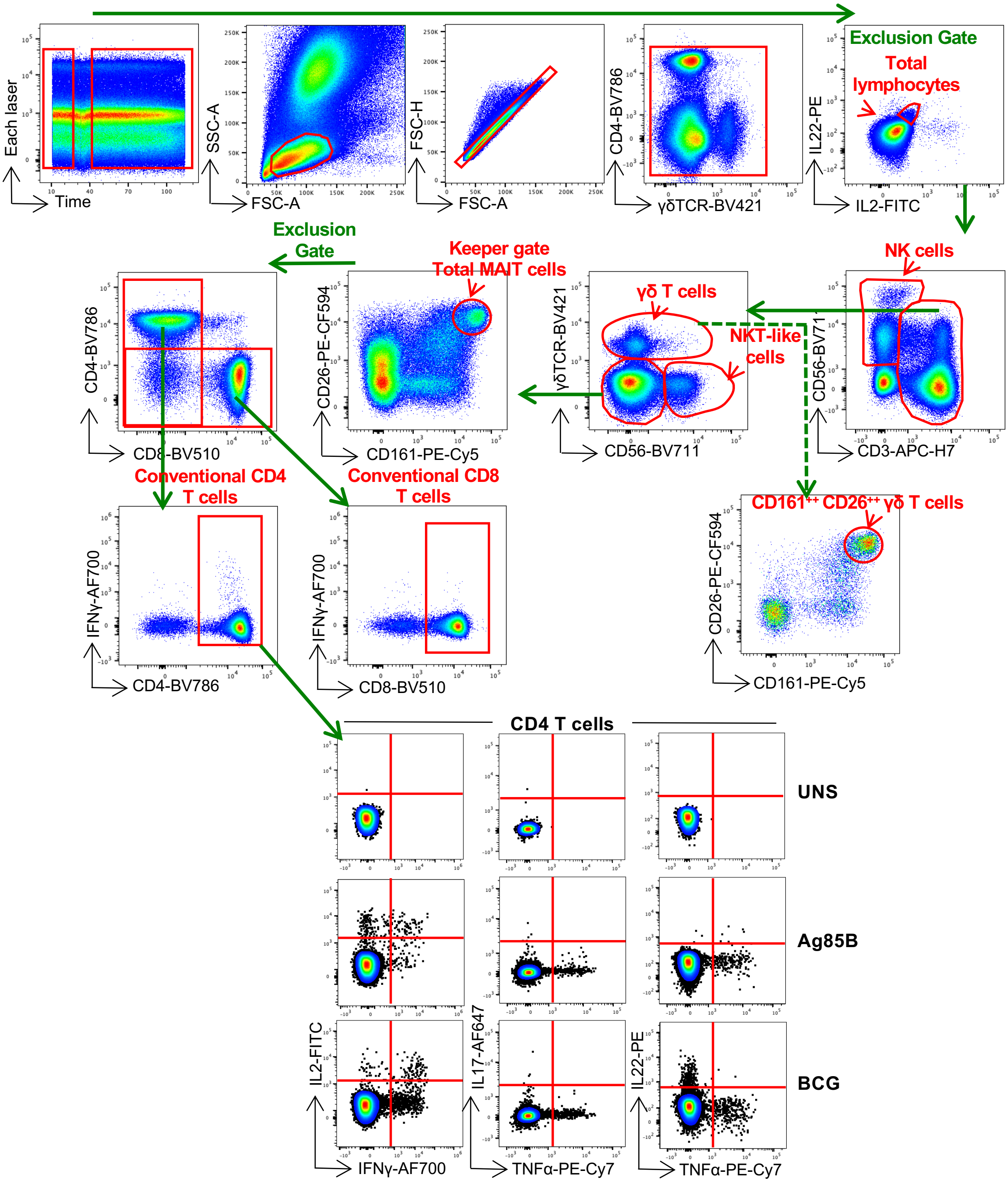

Expert gating strategy to identify conventional CD4 and CD8 T cells as well as donor-unrestricted T cells and NK cells and their cytokine expression in stimulated whole blood. This gating strategy is used in the same way to gate on cytokine producing immune cells (NK cells, **γδ** T cells, NKT_{like} cells, phenotypic MAIT, conventional CD4 and CD8 T cells, CD161+CD26+ **γδ** T cells) for functional analysis and unsupervised methods as well as to validate subset identification by unsupervised methods. In the latter, the gating strategy is applied on concatenated samples used to perform the tSNE analysis.

Supplementary Figure 3. Ag85B and TB10.4 specific immune responses post- vaccination with H4:IC31.

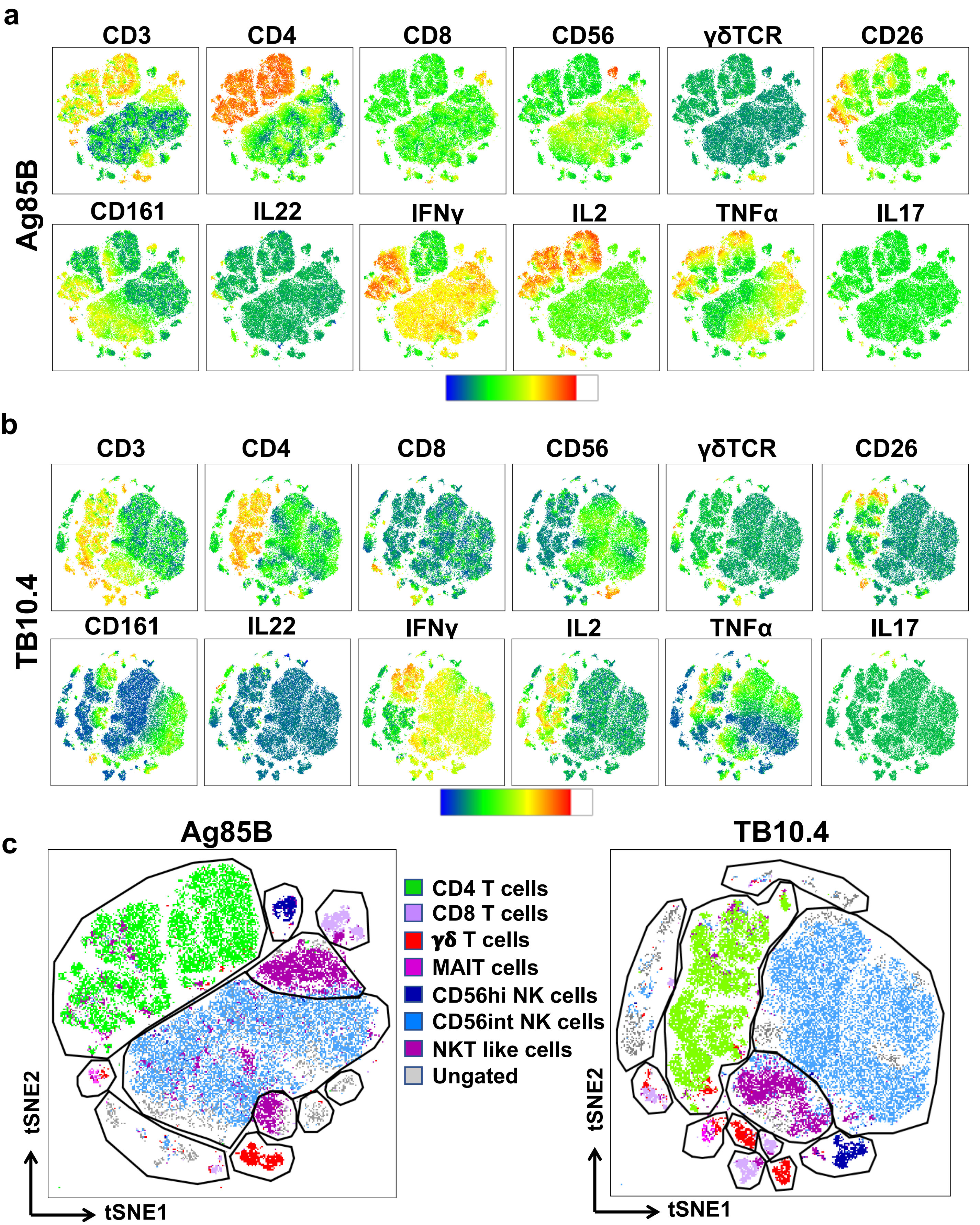

Multi-dimensional single cell events are shown in two-dimensional plots with overlay of the median intensity of marker expression from the lowest (blue) to the highest (red) to allow identification of the different cell clusters. Marker identification is shown for Ag85B total response **(a)** as well as for TB10.4 **(b).** In **c** are shown the overlays of manually gated phenotypic immune subsets identified on the total Ag85B (left) and TB10.4 (right) over the tSNE maps obtained for either stimulation, respectively. This analysis confirms tSNE performance to accurately identify subsets as compared to the gold standard (expert manual gating) and shows identification of cell populations overlooked by manual gating.

Supplementary Figure 4: Functions of immune subsets responding to Ag85B and TB10.4.

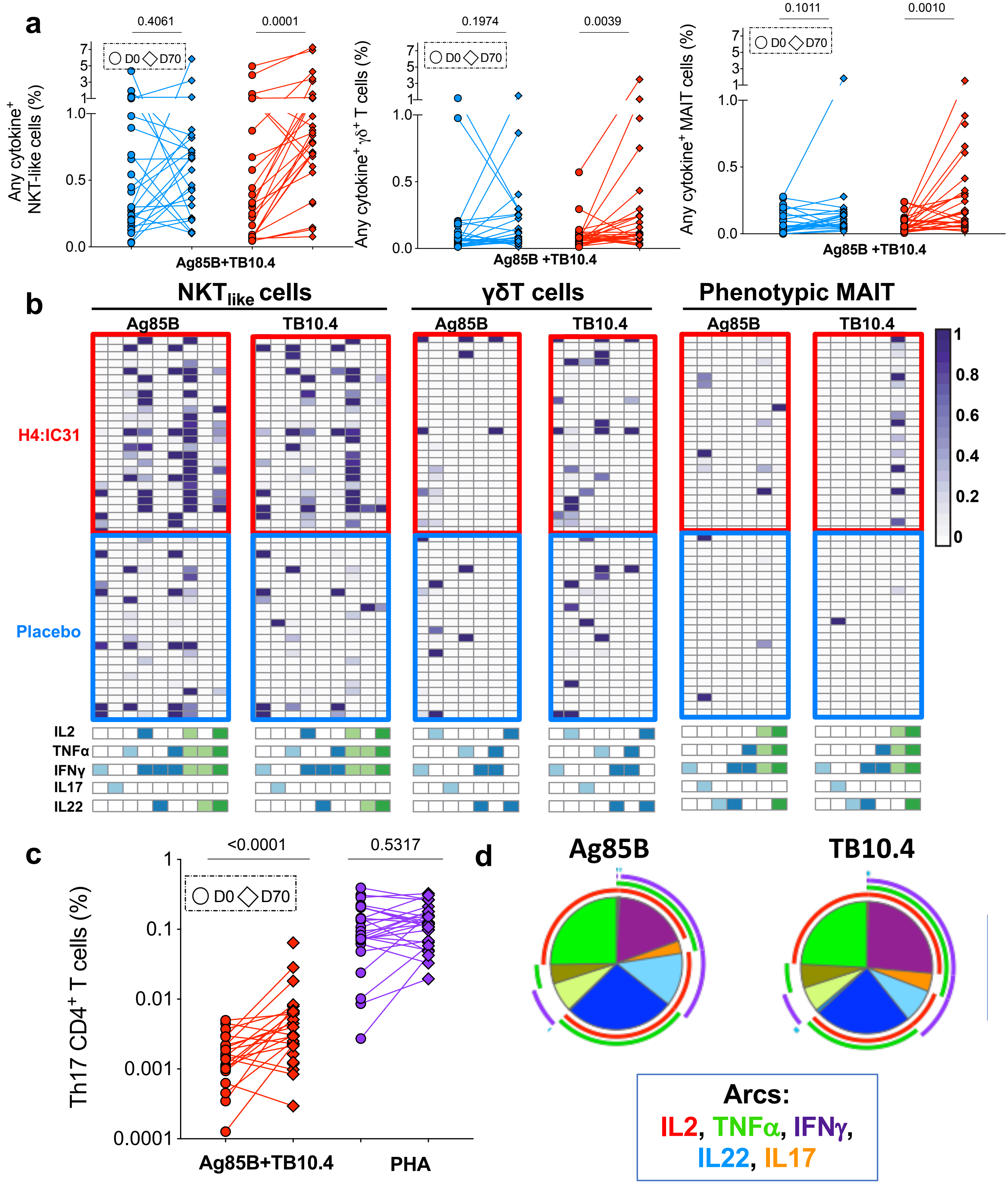

(a) NKT_{like} cell, γδ T cell and MAIT cell responses measured by whole blood intracellular cytokine staining (ICS) and flow cytometry following stimulation with Ag85B or TB10.4 peptide pools (summed response is shown). Shown are paired responses of immune cells expressing any combination of IFNγ, IL2, TNF, IL22 and/or IL17 for each individual at day 0 (circles) and day 70 (diamonds) randomized to placebo (blue) or H4:IC31 (red) arms. Changes in response between day 0 and day 70 were calculated by Wilcoxon Signed-Rank Test. **(b)** Heatmap of COMPASS posterior probabilities for day 70 response over day 0 in NKT_{like}, γδT cells and MAIT cells represents functional changes induced by vaccine or placebo administration. Columns show different cell subsets (shown are the 9, 7 and 7 of 24 subsets with detectable antigen-specific responses in at least 1 participant regardless of antigen specificity in NKT_{like}, γδ T cells and MAIT cells, respectively), color-coded by the cytokines they express (white=none, shaded=present) and ordered by degree of functionality from one function on the left to five functions on the right. Rows correspond to subjects ordered by vaccine arm. Each cell shows the probability that the corresponding cell subset (column) exhibits an Ag-specific response in the corresponding subject (row), where the probability is color-coded from white (zero) to purple (one). **(c)** Pies represent the total antigen-specific CD4 response (cells producing any combination of cytokines). Median relative contribution of cells co-expressing the cytokines identified by arcs is proportional to each slice size. CD4 polyfunctional profiles in response to Ag85B or TB10.4 stimulation in participants from H4:IC31 arm at day 70 were not significantly different (permutation test). **(d)** Th17 responses measured by whole blood intracellular cytokine staining (ICS) and flow cytometry following stimulation with Ag85B or TB10.4 peptide pools (summed response is shown) or PHA. Shown are paired responses of T cells expressing IL-17 for each individual at day 0 (circles) and day 70 (diamonds) in H4:IC31 arm. Changes in response between day 0 and day 70 were calculated by Wilcoxon Signed-Rank.

Supplementary Figure 5: Immune responses induced by H4:IC31 or BCG revaccination.

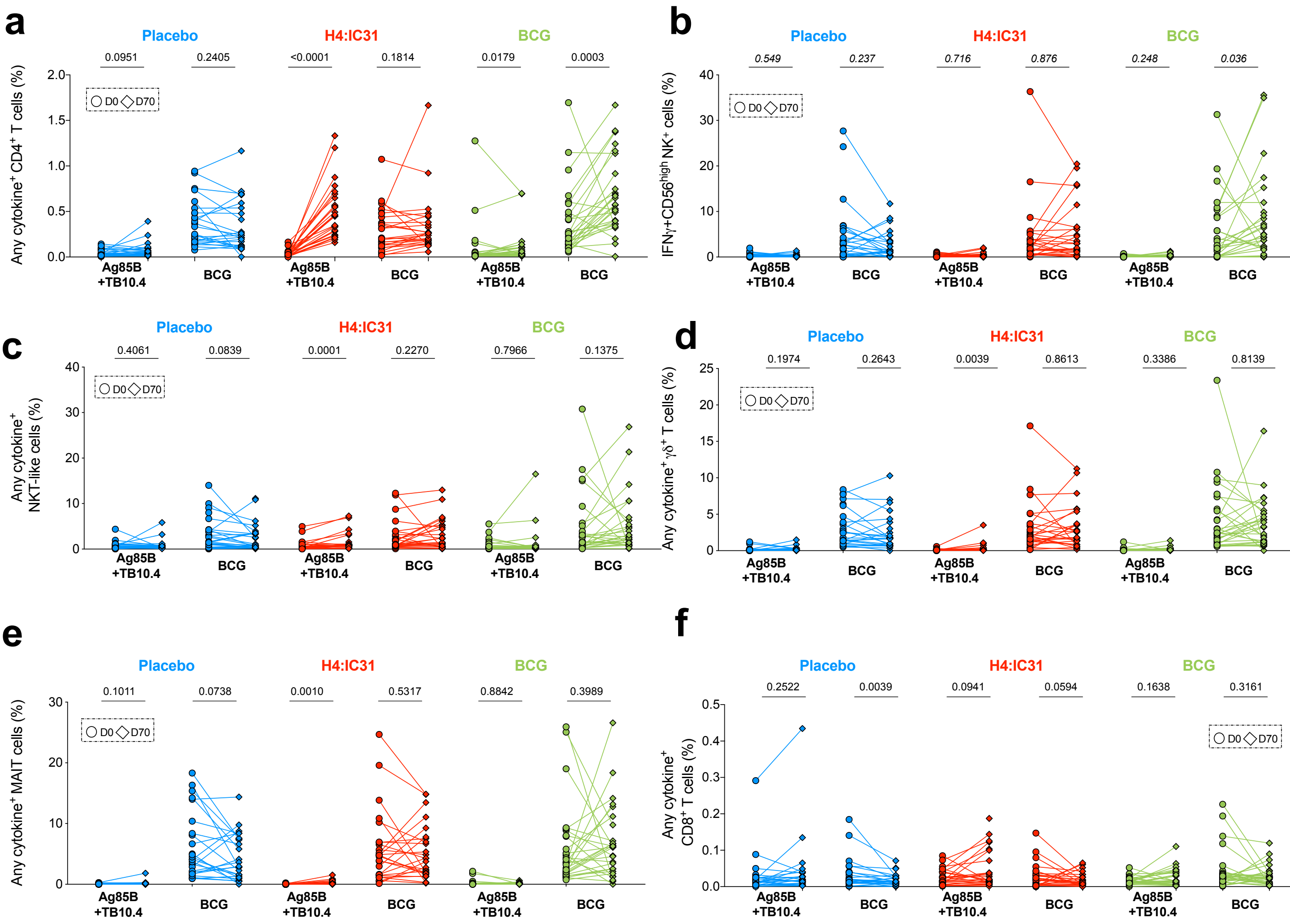

Vaccine immunogenicity was measured by whole blood intracellular cytokine staining (ICS) and flow cytometry following stimulation of whole blood with Ag85B or TB10.4 peptide pools (summed response is shown) or BCG. Paired responses of CD4 T cells **(a)**, IFN+CD56high NK cells **(b)**, NKT_{like} cells **(c)**, γδ T cells **(d),** MAIT cells **(e)** and CD8 T cells **(f)**, expressing any combination of IFNγ, IL-2, TNF, IL-22 and/or IL-17 for each individual at day 0 (circles) and day 70 (diamonds) randomized to placebo (blue), H4:IC31 (red) or BCG (green). Changes in response between day 0 and day 70 were calculated by Wilcoxon Signed-Rank Test.

Supplementary Figure 6: Immune responses to BCG restimulation.

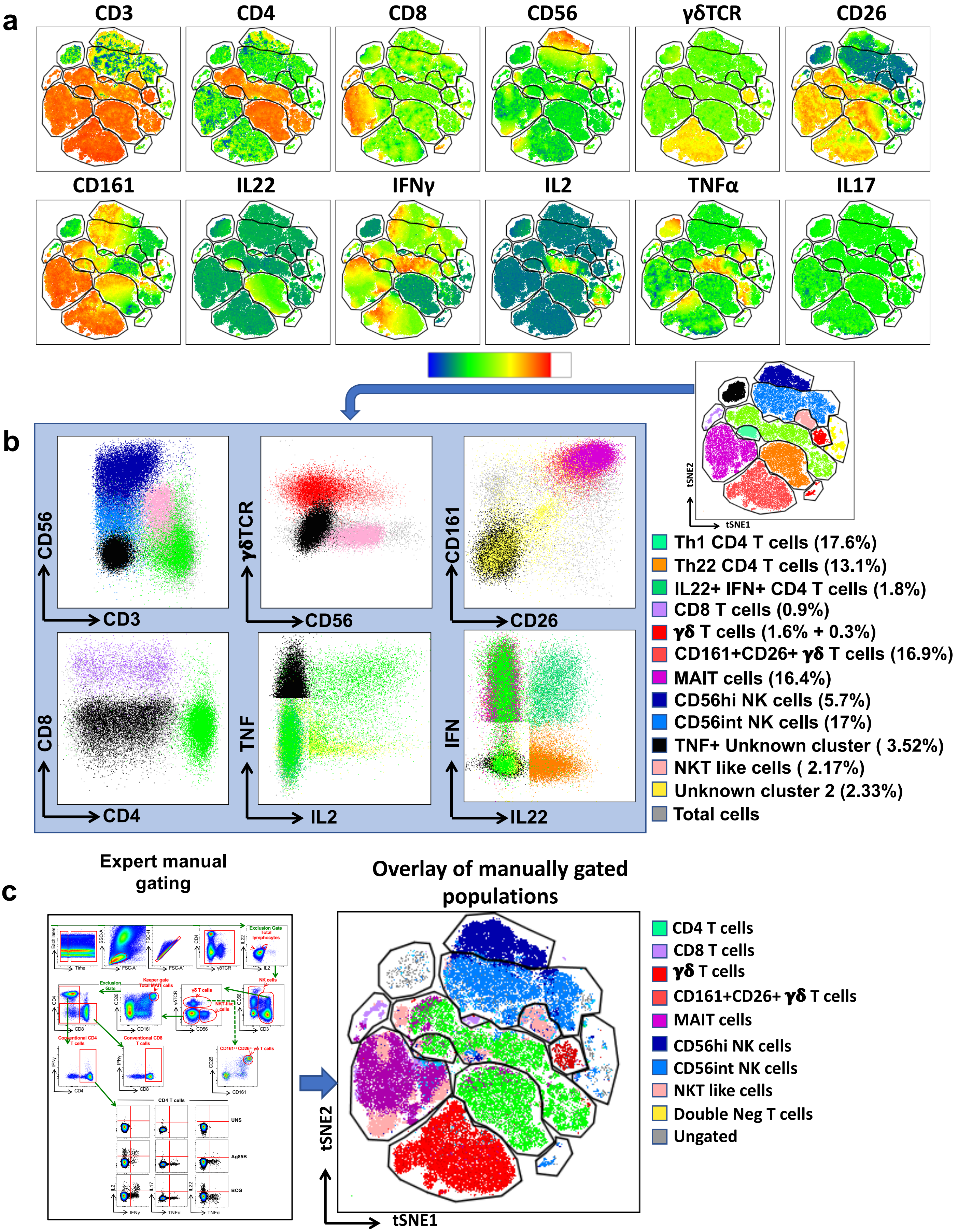

tSNE analysis of the total lymphocyte response observed in participants from all study arms at day 70 after whole blood re-stimulation with BCG and analyzed by flow cytometry. **(a)** Marker expression plots representing the multi-dimensional single cell events shown in two-dimensional plots with overlay of the median intensity of marker expression from the lowest (blue) to the highest (red) to allow identification of the different cell clusters. Marker expression plots allowed the identification of Th1, Th22 and other conventional CD4 or CD8 T cells, $CD26^{hi}CD161^{hi}$ phenotypic MAITs, γδ T cells, NK and NKT_{like} cell subsets, as well as unknown cell subsets (partly ungated) for which we did not include appropriate phenotypic markers in the flow cytometry panel (Figure 3A). **(b)** Subsets identified by the tSNE map were individually overlaid on biplot with all combinations of markers present in our flow cytometry panel to validate the accuracy of the clustering performed by the tSNE analysis. In **(c)** are shown the overlays of manually gated immune subsets identified on the total BCG response over the tSNE map. This analysis **(b and c)** confirms tSNE performance to accurately identify subsets as compared to the gold standard (expert manual gating), and shows identification of cell populations overlooked by manual gating.

Supplementary Figure 7: Differentially expressed immune subsets in the context of BCG revaccination.

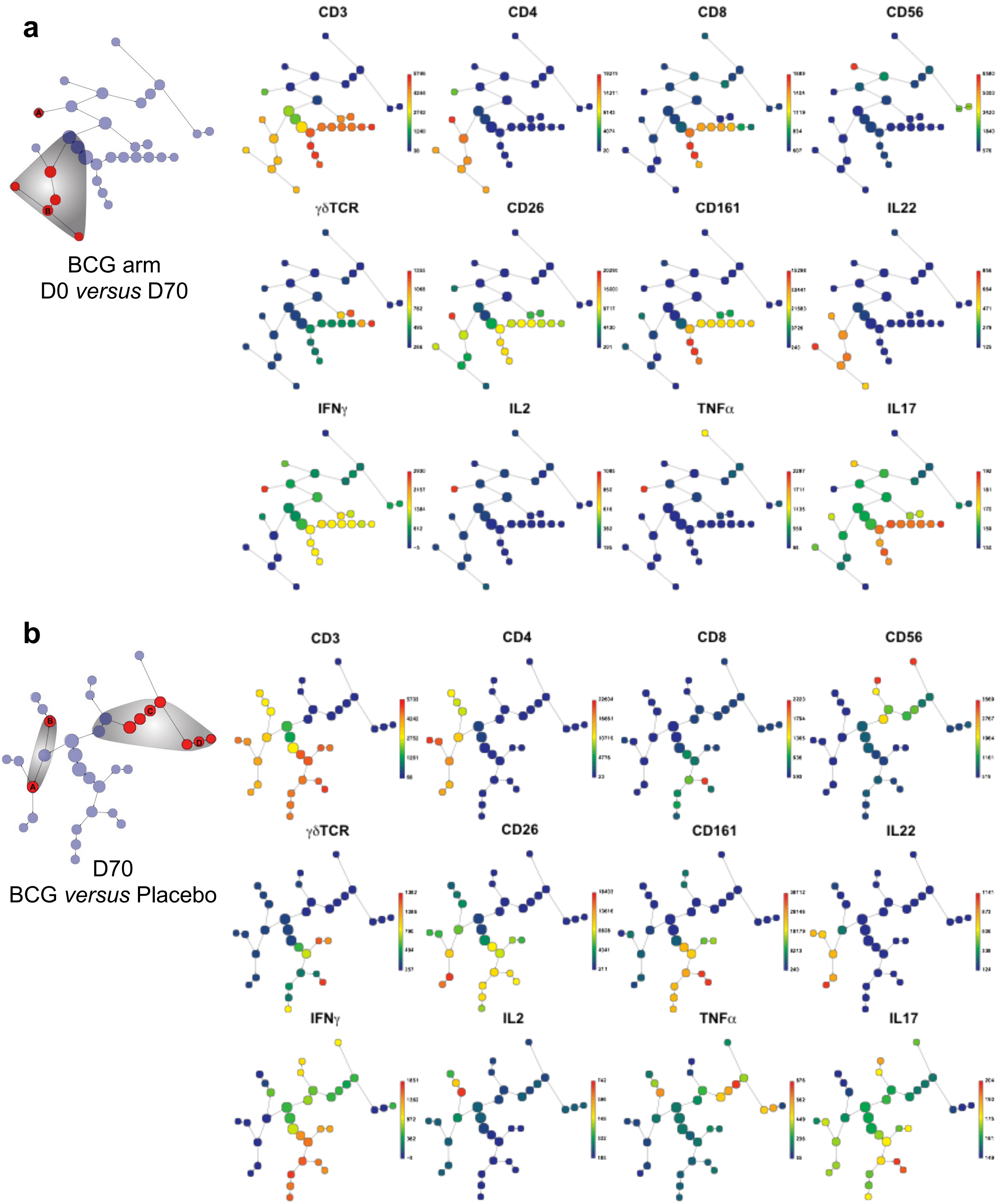

(a) Visual representation of CITRUS unsupervised hierarchical clustering of any cytokine-producing lymphocytes (Supplementary Figure 1) after BCG-restimulation comparing day 0 and day 70 in the BCG arm. Differentially expressed clusters based on SAM analysis (FDR <0.1) are circled in gray and highlighted in red. Expression levels of each marker are color-coded and overlaid on the CITRUS tree to identify cluster identity. **(b)** CITRUS unsupervised hierarchical clustering measured at day 70 in placebo *versus* BCG arm. Expression levels of each marker are color-coded and overlaid on the CITRUS tree to identify cluster identity.

Supplementary Figure 8: Functions of BCG induced immune responses.

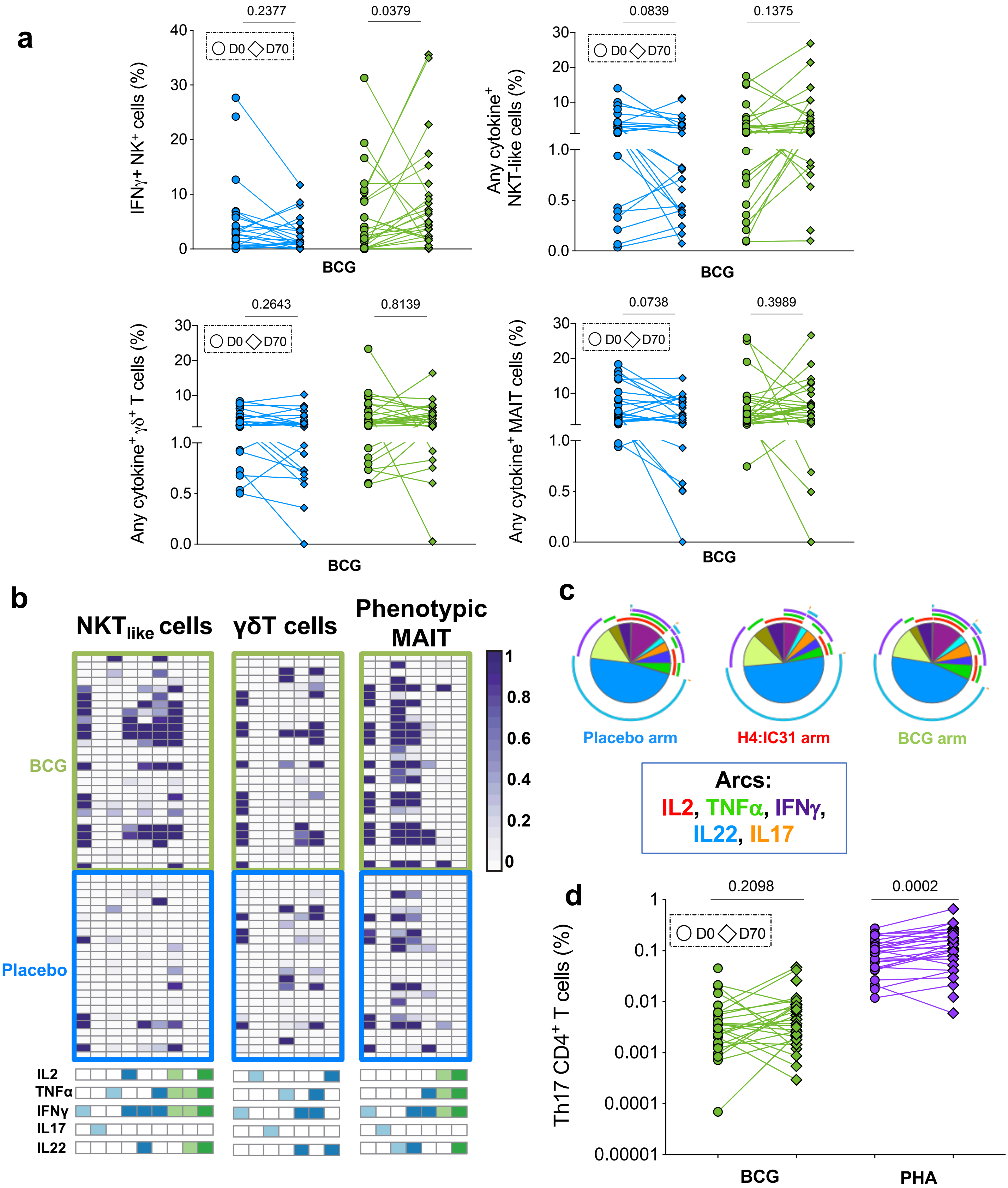

(a) NK, NKT$_{like}$, γδ T cell and MAIT cell responses measured by whole blood intracellular cytokine staining (ICS) and flow cytometry following stimulation with BCG. Shown are paired responses of T cells expressing any combination of IFNγ, IL-2, TNF, IL-22 and/or IL-17 for each individual at day 0 (circles) and day 70 (diamonds) randomized to placebo (blue) or BCG (green) arms. Changes in response between day 0 and day 70 were calculated by Wilcoxon Signed-Rank Test. **(b)** Heatmap of COMPASS posterior probabilities for day 70 response over day 0 in NKT-like, γδ T cells and MAIT cells represents functional changes induced by BCG vaccine or placebo administration. Columns show different cell subsets (shown are the 9, 7 and 7 of 24 subsets with detectable antigen-specific responses in at least 1 participant regardless of antigen specificity in NKT$_{like}$, γδ T cells and MAIT cells, respectively), color-coded by the cytokines they express (white=none, shaded=present) and ordered by degree of functionality from one function on the left to five functions on the right. Rows correspond to subjects ordered by vaccine arm. Each cell shows the probability that the corresponding cell subset (column) exhibits a BCG-specific response in the corresponding subject (row), where the probability is color-coded from white (zero) to purple (one). **(c)** Pies represent the total antigen specific CD4 response (cells producing any combination of cytokines). Median relative contribution of cells co-expressing the cytokines identified by arcs is proportional to each slice size. CD4 polyfunctional profiles in response to BCG stimulation in participants from placebo, H4:IC31, or BCG arm at day 70 were not significantly different (permutation test). **(d)** Th17 responses measured by whole blood intracellular cytokine staining (ICS) and flow cytometry following stimulation with BCG or PHA. Shown are paired responses of T cells expressing IL-17 for each individual at day 0 (circles) and day 70 (diamonds) in BCG arm. Changes in response between day 0 and day 70 were calculated by Wilcoxon Signed-Rank.

Supplementary Figure 9: Vaccine responders.

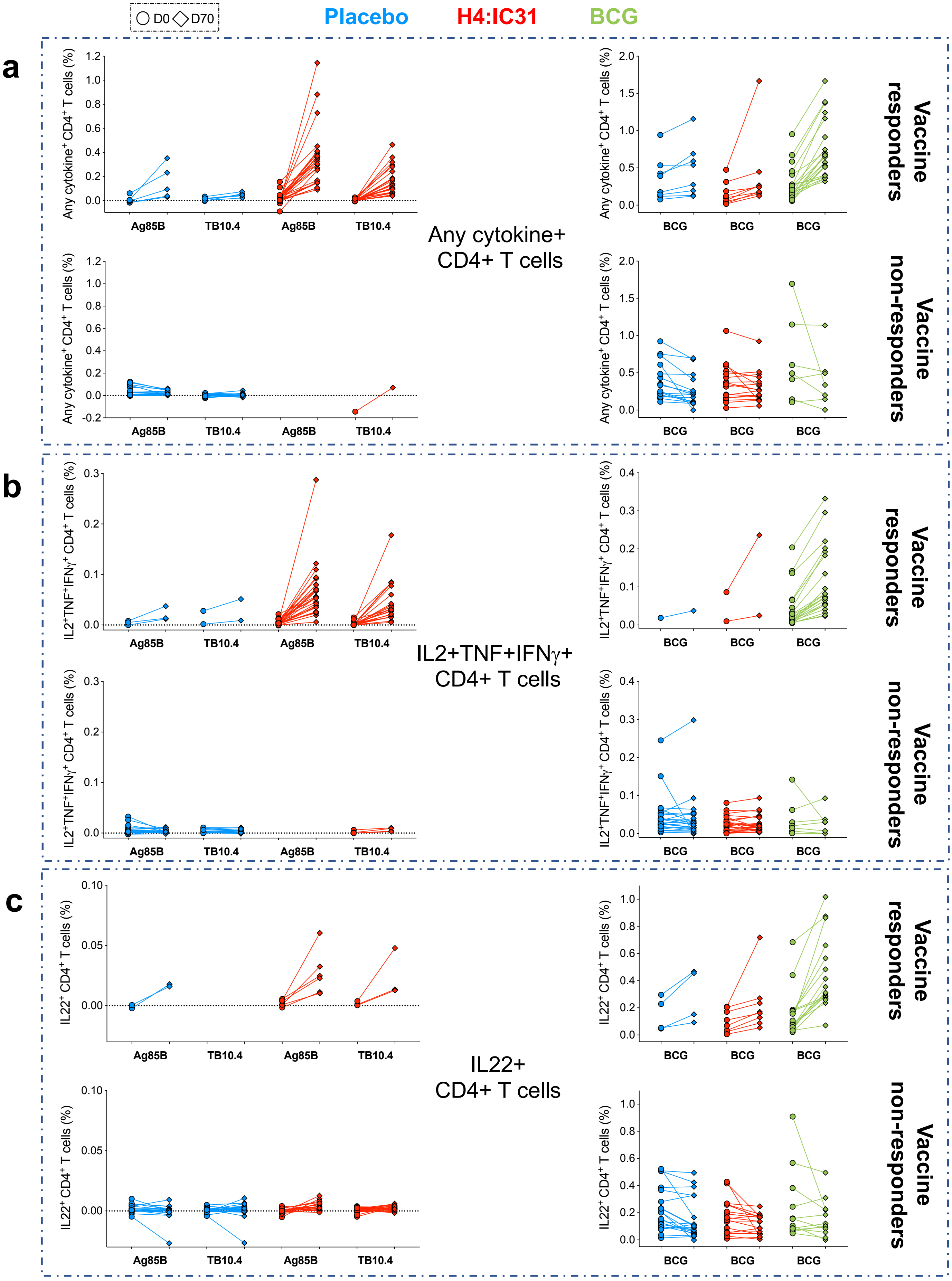

Vaccine immunogenicity measured by whole blood intracellular cytokine staining (ICS) and flow cytometry following stimulation with Ag85B or TB10.4 peptide pools (left panels) and BCG (right panels) stratified by responder status (calculated by MIMOSA2) and vaccine arms (placebo (blue), H4:IC31 (red) and BCG (green). Frequencies of CD4 T cells expressing any combination of IFNγ, IL-2, TNF, IL-22 and/or IL-17 **(a)**, polyfunctional IFNγ+ IL-2+ TNF+ **(b)** and IL-22+ **(c)** are shown for responders and non-responders at day 0 (circles) and day 70 (diamonds).

Supplementary Table 1: Antibody panels used to perform the PBMC (a) and whole blood (b) ICS assays.

a

Marker	Fluorochrome	Clone	Supplier	Rationale
CD3	ECD	UCHT1	Beckman Coulter	Lineage
CD4	APC-eFluor780	RPA-T4	eBioscience	
CD8	AF700	HIT8a	BioLegend	
CD45RO	BV785	UCHL1	BioLegend	Memory
CCR7	BV605	G043H7	BioLegend	
IL2	PE	MQ1-17H12	BD	Function
IFNg	V450	B27	BD	
TNF	PE-Cy7	MAb11	BD	
IL17A	PerCP-Cy5.5	BL168	BioLegend	
IL22	APC	IL22JOP	eBioscience	
CD154	PE-Cy5	TRAP1	BD	
CD107a	AF488	H4A3	BioLegend	
CD14	V500	M5E2	BD	Dump
CD19	V500	HIB19	BD	
Live/Dead	Aqua	L34957	Life Technologies	

b

Marker	Fluorochrome	Clone	Supplier	Rationale
CD3	APC-H7	SK7	BD	Lineage
CD4	BV786	SK3	BD	
CD8	BV510	SK1	BD	
γδ TCR	BV421	B1	Biolegend	
CD56	BV711	HCD56	Biolegend	
CD161	PE-CY5	DX12	BD	
CD26	PE-CF594	M-A261	BD	
IL2	FITC	5344.111	BD	Function
IFNγ	AF700	B27	BD	
TNFα	PE-CY7	Mab11	eBioscience	
IL17	AF647	SCPL1362	BD	
IL22	PE	22URTI	eBioscience	

Supplementary Table 2: Demographic characteristics of the immunogenicity cohort.

Vaccine arm			Placebo	H4:IC31	BCG
**n**			24	26	28
**Age**		median (min-max)	15 (13-17)	14 (12-16)	14 (12-16)
**Gender**		% male	50	29.6	57.1
**Ethnicity**					
	Coloured	n (%)	22 (91.7)	22 (84.6)	25 (89.3)
	Black	n (%)	2 (8.3)	4 (15.4)	3 (10.7)
**BMI**		median (min-max)	19.95 (16.4-24.8)	19.2 (17-27)	20.15 (15.5-27.2)
