# Supplementary material for: Multidimensional analyses reveal modulation of adaptive and innate immune subsets by tuberculosis vaccines
Source: Commun Biol. 2020 Oct 9;3:563. doi: 10.1038/s42003-020-01288-3 (PMC7547090; doi:10.1038/s42003-020-01288-3)
Supplement: Supplementary file 2 — Reporting Summary [file 42003_2020_1288_MOESM2_ESM.pdf]

nature research

Corresponding author(s): Virginie Rozot, Thomas J. Scriba

Last updated by author(s): 10th June 2020

Reporting Summary

Nature Research wishes to improve the reproducibility of the work that we publish. This form provides structure for consistency and transparency in reporting. For further information on Nature Research policies, see our Editorial Policies and the Editorial Policy Checklist.

Statistics

For all statistical analyses, confirm that the following items are present in the figure legend, table legend, main text, or Methods section.

n/a	Confirmed	
☐	☒	The exact sample size (n) for each experimental group/condition, given as a discrete number and unit of measurement
☐	☒	A statement on whether measurements were taken from distinct samples or whether the same sample was measured repeatedly
☐	☒	The statistical test(s) used AND whether they are one- or two-sided<br>*Only common tests should be described solely by name; describe more complex techniques in the Methods section.*
☒	☐	A description of all covariates tested
☒	☐	A description of any assumptions or corrections, such as tests of normality and adjustment for multiple comparisons
☐	☒	A full description of the statistical parameters including central tendency (e.g. means) or other basic estimates (e.g. regression coefficient) AND variation (e.g. standard deviation) or associated estimates of uncertainty (e.g. confidence intervals)
☐	☒	For null hypothesis testing, the test statistic (e.g. F, t, r) with confidence intervals, effect sizes, degrees of freedom and P value noted<br>*Give P values as exact values whenever suitable.*
☒	☐	For Bayesian analysis, information on the choice of priors and Markov chain Monte Carlo settings
☒	☐	For hierarchical and complex designs, identification of the appropriate level for tests and full reporting of outcomes
☒	☐	Estimates of effect sizes (e.g. Cohen's d, Pearson's r), indicating how they were calculated

Our web collection on statistics for biologists contains articles on many of the points above.

Software and code

Policy information about availability of computer code

Data collection	N/A
Data analysis	COMPASS1 R package, MIMOSA2 R package, GraphPad Prism v7.0c, FlowJo 10.6.1, Cytobank

For manuscripts utilizing custom algorithms or software that are central to the research but not yet described in published literature, software must be made available to editors and reviewers. We strongly encourage code deposition in a community repository (e.g. GitHub). See the Nature Research guidelines for submitting code & software for further information.

Data

Policy information about availability of data

All manuscripts must include a data availability statement. This statement should provide the following information, where applicable:

- Accession codes, unique identifiers, or web links for publicly available datasets
- A list of figures that have associated raw data
- A description of any restrictions on data availability

Immunogenicity data from the C-040-404 clinical trial are collated in .csv format on Figshare (DOI 10.25375/uct.12472739).

Field-specific reporting

Please select the one below that is the best fit for your research. If you are not sure, read the appropriate sections before making your selection.

☒ Life sciences ☐ Behavioural & social sciences ☐ Ecological, evolutionary & environmental sciences

For a reference copy of the document with all sections, see nature.com/documents/nr-reporting-summary-flat.pdf

Life sciences study design

All studies must disclose on these points even when the disclosure is negative.

Sample size	The first 90 sequentially enrolled participants, approximately 30 per arm, were included in the immunogenicity and safety cohort from the C-040-404 phase IIb, randomized, three-arm, placebo-controlled, partially-blinded clinical trial conducted at two South African sites reported in the present manuscript.
Data exclusions	4 sample sets were excluded from the whole blood ICS assay dataset because they did not meet the following inclusion criteria: : a) unstimulated control was present and interpretable for each set of samples; b) frequencies of PHA- or BCG-stimulated total cytokine-expressing CD4 or CD8 T cells were greater than the median + 3MAD (median absolute deviation) of the total cytokine+ CD4 or CD8 T cells of the unstimulated controls of the entire cohort; c) for each sample, the frequency of PHA- or BCG-stimulated total cytokine+ CD4 or CD8 T cells were greater than the frequency of the same cell population in its respective unstimulated control.
Replication	The reproducibility of findings my different analysis strategies is presented in this manuscript are summarized in figure 1. Unsupervised analyses were backed up by manual gating of the identified population. This is shown in supplementary figures 3 and 6.
Randomization	Eligible participants were randomized at a 1:1:1 ratio to receive intramuscular placebo (saline) or H4:IC31 (15ug H4 polyprotein composed of Ag85B and TB10.4 Mtb proteins, Sanofi Pasteur, in 500nmol IC31 adjuvant, Valneva, Vienna, Austria) on day 0 and day 56, or intradermal BCG (2-8 x 105 CFU, Statens Serum Institut) at day 0.
Blinding	Data were exported, locked and analyzed prior to unblinding. For tSNE and CITRUS analyses data were unblinded to assign participant data to the different vaccine arms.

Reporting for specific materials, systems and methods

We require information from authors about some types of materials, experimental systems and methods used in many studies. Here, indicate whether each material, system or method listed is relevant to your study. If you are not sure if a list item applies to your research, read the appropriate section before selecting a response.

Materials & experimental systems

n/a	Involved in the study	
☐	☒	Antibodies
☒	☐	Eukaryotic cell lines
☒	☐	Palaeontology and archaeology
☒	☐	Animals and other organisms
☐	☒	Human research participants
☐	☒	Clinical data
☒	☐	Dual use research of concern

Methods

n/a	Involved in the study	
☒	☐	ChIP-seq
☐	☒	Flow cytometry
☒	☐	MRI-based neuroimaging

Antibodies

Antibodies used	Antibodies used, fluorochromes, clones and supplier are listed in Supplementary tables 2A and 2B.
Validation	Optimization of flow cytometry panels includes titration of all reagents, “fluorescence minus one” experiments, comparison with reference reagents used in previous assays if applicable, testing of relevant samples prior to use in the target populations.

Human research participants

Policy information about studies involving human research participants

Population characteristics	Number of participants, age, gender, ethnicity and BMI are presented per trial arm (Placebo, H4:IC31 and BCG) in supplementary table 1.
Recruitment	Participants were recruited from high schools or from the community at the South African Tuberculosis Vaccine Initiative (SATVI) Worcester research site and at the Emavundleni Research Centre in Nyanga, Western Cape Province of South Africa,

	where community members have historically been enthusiastically engaged in TB clinical research.<br>Individual participant assent was obtained after consent by the parent or legal guardian prior to screening.
Ethics oversight	The protocol was approved by the South African Health Products Regulatory Authority (SAHPRA), formerly the Medicines Control Council of South Africa (Reference number: 20130826) and the Human Research Ethics Committee of the University of Cape Town (SATVI site reference: 471/2013; Emavundleni site reference: 615/2014).

Note that full information on the approval of the study protocol must also be provided in the manuscript.

Clinical data

Policy information about clinical studies

All manuscripts should comply with the ICMJE guidelines for publication of clinical research and a completed CONSORT checklist must be included with all submissions.

Clinical trial registration	ClinicalTrials.gov number, NCT02075203
Study protocol	Available in the supplement of the primary publication of the C-040-404 trial: https://www.nejm.org/doi/full/10.1056/NEJMoa1714021
Data collection	Immunology data presented in this paper were generated from March 29, 2016 to May 31, 2016 on samples collected from participants of the C-040-404 trial, enrolled between April 1, 2014, and May 25, 2015, at two sites in South Africa.
Outcomes	Outcomes described in this paper represent exploratory immunology outcomes from the immunogenicity sub-cohort of the C-040-404 trial. All primary flow cytometry analyses were conducted by laboratory personnel who were blinded to study group allocation.

Flow Cytometry

Plots

Confirm that:

☒ The axis labels state the marker and fluorochrome used (e.g. CD4-FITC).

☒ The axis scales are clearly visible. Include numbers along axes only for bottom left plot of group (a 'group' is an analysis of identical markers).

☒ All plots are contour plots with outliers or pseudocolor plots.

☒ A numerical value for number of cells or percentage (with statistics) is provided.

Methodology

Sample preparation	Venous blood was collected on day 0 and day 70 in sodium heparin-containing tubes and processed for analysis of antigen-specific cell responses by whole blood ICS assay. Briefly, within 75 minutes of collection, whole blood was stimulated in presence of co-stimulatory antibodies (anti-CD28 and anti-CD49d, BD Biosciences, 0.25 µg/mL each) with medium alone (unstimulated, RPMI, Lonza), Ag85B peptide pool (15 mers overlapping by 11 amino acids, JPT, 2µg/mL/peptide), TB10.4 (15 mers overlapping by 11 amino acids, Aeras, 2µg/mL/peptide), BCG vaccine (1.2x106 CFU/mL, Statens Serum Institute), or PHA (Phytohemagglutinin as positive control, 5µg/mL, Bioweb). Following 7 hours of incubation at 37°C, Brefeldin A (10µg/mL, Sigma Aldrich) was added and blood was incubated for an additional 5 hours. Thereafter, blood was incubated with EDTA (2mM, Sigma-Aldrich), red blood cells were lysed, and white blood cells fixed using FACSlysing solution (BD Biosciences) prior to cryopreservation in a solution of RPMI, 40% Fetal Calf Serum (FCS, Hyclone) and 10% DMSO (Sigma Aldrich).<br>After thawing, cells were permeabilized for 10 minutes at room temperature in BD Perm/Wash and stained with a panel of pre-titrated monoclonal antibodies diluted in Brilliant Staining buffer (BD Biosciences) for 45-60 minutes at 4°C. Compensation for each experiment was calculated from single-stained antibody capture beads (BD Biosciences) acquired with the samples on the flow cytometer.
Instrument	BD Fortessa flow cytometer, equipped with 4 lasers (405nm, 488nm, 544nm, 633nm) and 18 detectors for fluorescent parameters.
Software	FlowJo 10.6.1
Cell population abundance	*Describe the abundance of the relevant cell populations within post-sort fractions, providing details on the purity of the samples and how it was determined.*
Gating strategy	The expert gating strategy to identify conventional CD4 and CD8 T cells as well as donor-unrestricted T cells and NK cells and their cytokine expression in stimulated whole blood is shown in supplementary Figure 1. This gating strategy is used in the same way to gate on cytokine producing immune cells (NK cells, gdT cells, NKTlike cells, phenotypic MAIT, conventional CD4 and CD8 T cells, CD161+CD26+ gdT cells) for functional analysis and unsupervised methods as well as to validate subset identification by unsupervised methods. In the latter, the gating strategy is applied on concatenated samples used to perform the tSNE analysis.

☒ Tick this box to confirm that a figure exemplifying the gating strategy is provided in the Supplementary Information.
